# Vaccine Take of RV3-BB Rotavirus Vaccine Observed in Indonesian Infants Regardless of HBGA Status

**DOI:** 10.1093/infdis/jiad351

**Published:** 2023-08-18

**Authors:** Celeste M Donato, Amanda Handley, Sean G Byars, Nada Bogdanovic-Sakran, Eleanor A Lyons, Emma Watts, Darren S Ong, Daniel Pavlic, Jarir At Thobari, Cahya Dewi Satria, Hera Nirwati, Yati Soenarto, Julie E Bines

**Affiliations:** Enteric Diseases Group, Murdoch Children's Research Institute; Department of Paediatrics, The University of Melbourne, Parkville; Biomedicine Discovery Institute and Department of Microbiology, Monash University, Melbourne; Enteric Diseases Group, Murdoch Children's Research Institute; Medicines Development for Global Health, Southbank; Florey Institute of Neuroscience and Mental Health, Parkville, Australia; Enteric Diseases Group, Murdoch Children's Research Institute; Enteric Diseases Group, Murdoch Children's Research Institute; Enteric Diseases Group, Murdoch Children's Research Institute; Enteric Diseases Group, Murdoch Children's Research Institute; Enteric Diseases Group, Murdoch Children's Research Institute; Department of Pharmacology and Therapy; Center for Child Health; Department of Paediatrics; Center for Child Health; Department of Microbiology, Faculty of Medicine, Public Health and Nursing, Universitas Gadjah Mada; Center for Child Health; Department of Child Health, Faculty of Medicine, Public Health and Nursing, Universitas Gadjah Mada, Dr Sardjito Hospital, Yogyakarta, Indonesia; Enteric Diseases Group, Murdoch Children's Research Institute; Department of Paediatrics, The University of Melbourne, Parkville; Department of Gastroenterology and Clinical Nutrition, Royal Children's Hospital, Parkville, Australia

**Keywords:** histo-blood group antigen, Indonesia, neonatal vaccination, rotavirus, vaccine take

## Abstract

**Background:**

Histo-blood group antigen (HBGA) status may affect vaccine efficacy due to rotavirus strains binding to HBGAs in a P genotype–dependent manner. This study aimed to determine if HBGA status affected vaccine take of the G3P[6] neonatal vaccine RV3-BB.

**Methods:**

DNA was extracted from stool samples collected in a subset (n = 164) of the RV3-BB phase IIb trial in Indonesian infants. *FUT2* and *FUT3* genes were amplified and sequenced, with any single-nucleotide polymorphisms analyzed to infer Lewis and secretor status. Measures of positive cumulative vaccine take were defined as serum immune response (immunoglobulin A or serum-neutralizing antibody) and/or stool excretion of RV3-BB virus. Participants were stratified by HBGA status and measures of vaccine take.

**Results:**

In 147 of 164 participants, Lewis and secretor phenotype were determined. Positive vaccine take was recorded for 144 (97.9%) of 147 participants with the combined phenotype determined. Cumulative vaccine take was not significantly associated with secretor status (relative risk, 1.00 [95% CI, .94–1.06]; *P* = .97) or Lewis phenotype (relative risk, 1.03 [95% CI, .94–1.14]; *P* = .33), nor was a difference observed when analyzed by each component of vaccine take.

**Conclusions:**

The RV3-BB vaccine produced positive cumulative vaccine take, irrespective of HBGA status in Indonesian infants.

## Teaser

Population differences in histo-blood group antigen status may contribute to the varying performance of oral rotavirus vaccines. This study revealed that the RV3-BB vaccine produced positive cumulative vaccine take, irrespective of histo-blood group antigen status in Indonesian infants.

Rotavirus remains the most important cause of acute gastroenteritis in infants and young children worldwide [1]. A disparity in rotavirus vaccine effectiveness has been observed for Rotarix (GlaxoSmithKline) and RotaTeq (Merck & Co), with increased effectiveness demonstrated in high-income settings as compared with low-income settings [2–4]. The reasons for this disparity are poorly understood and likely multifactorial. Population differences in histo-blood group antigen (HBGA) status could be one contributing factor.

HBGAs are complex glycans expressed on the surface of many cell types and are present as free oligosaccharides in biological fluids [5]. The expression of secretor and Lewis HBGAs is determined genetically by polymorphisms of the *FUT2* and *FUT3* genes. *FUT2* (*Se*) encodes an α(1,2)-fucosyltransferase that controls the synthesis of the H antigen (the precursor of the ABO blood group antigens). The secretor (*Se*) phenotype is inherited in an autosomal dominant manner, with the nonsecretor (*se*) phenotype a recessive trait. Individuals who are homozygous for *FUT2*-null alleles do not express ABO antigens. The Lewis blood group phenotypes are determined by the *FUT2* and *FUT3* genes, with *FUT3* also exhibiting a functional dominant allele (*Le*) and a nonfunctional recessive allele (*le*). There are 2 major Lewis blood group antigens: Le(a) and Le(b). Those who are homozygous recessive (*le/le)* do not express Le(a) or Le(b) and are Lewis negative (Le(a−b−)). This rare phenotype is observed in 5% to 10% of the European population [6]. For those who are homozygous dominant (*Le/Le*) or heterozygous (*Le/le*), the expression of Le(a) and Le(b) is dictated by their secretor status. Secretors who are *Le* positive express the Le(a−b+) phenotype, while nonsecretors who are *Le* positive express the Le(a+b−) phenotype. The Le(a+b+) phenotype is also considered rare, as it is only observed transiently in very young children due to developmental immaturity in *FUT2* activity [7]. *FUT2* variants in individuals with weak activity (*Se^w^*) have been observed, and *Se^385^* (rs1047781) is the most common allele associated with this polymorphism, being particularly observed in Polynesian and East and Southeast Asian populations [7, 8]. The enzyme encoded by this allele has 2% to 5% of the activity of the wild type enzyme [9].

The virion surface protein VP4 denotes the P genotype and can be cleaved into 2 subunits, VP5* and VP8*, with the VP8* globular head containing the glycan receptor domain involved in viral attachment [10]. Increasing evidence suggests that Lewis and secretor status may affect susceptibility to wild type rotavirus infection and that strains bind to HBGAs in a P genotype–dependent manner [11–16]. Studies in diverse populations in Europe, Asia, and Africa have revealed that secretors and those who are Lewis positive are more susceptible to wild type P[8] infection [12, 14, 17–20].

Population differences in HBGA status may contribute to the varying performance of oral rotavirus vaccines. The immune response to a live oral rotavirus vaccine is based on stimulation of the gastrointestinal mucosa through recognition, binding, and subsequent replication of the vaccine strain. The natural immunity to P[8] strains afforded by Lewis-negative and nonsecretor status may render infants unable to mount a robust immune response to P[8]-based vaccines. Lower effectiveness of P[8]-based vaccines has been reported, particularly in African countries where Lewis-negative and nonsecretor phenotypes predominate [18, 21–23]. The limited studies published to date investigating HBGA phenotype and rotavirus vaccine seroconversion or vaccine effectiveness have reported variable results [13, 20, 24–27].

The association between wild type P[6] strains and HBGA status is poorly understood. Studies in Burkina Faso, Korea, and Bangladesh revealed that P[6] strains predominantly infected individuals who were Lewis negative [18, 28, 29]. A study in South Africa reported a similar frequency of P[6] strains between secretors and nonsecretors [15]. It is not known if the structural differences between wild type and neonatal P[6] may further affect binding to HBGAs. Therefore, it is important to understand if the performance of non-P[8] vaccines, such as the neonatal P[6]-based vaccine RV3-BB, are affected by HBGA status.

The RV3-BB rotavirus vaccine is composed of a naturally attenuated human neonatal G3P[6] rotavirus strain [30]. Safety and immunogenicity of the vaccine were established in phase I and IIa trials conducted in Australia and New Zealand (NZ), respectively [31, 32]. A phase IIb trial for efficacy, safety, and immunogenicity was subsequently conducted in Indonesia [33]. In this study, we sought to determine whether Lewis and secretor status affected vaccine take after vaccination with RV3-BB.

## METHODS

### Study Design and Participants

**Figure 1.**
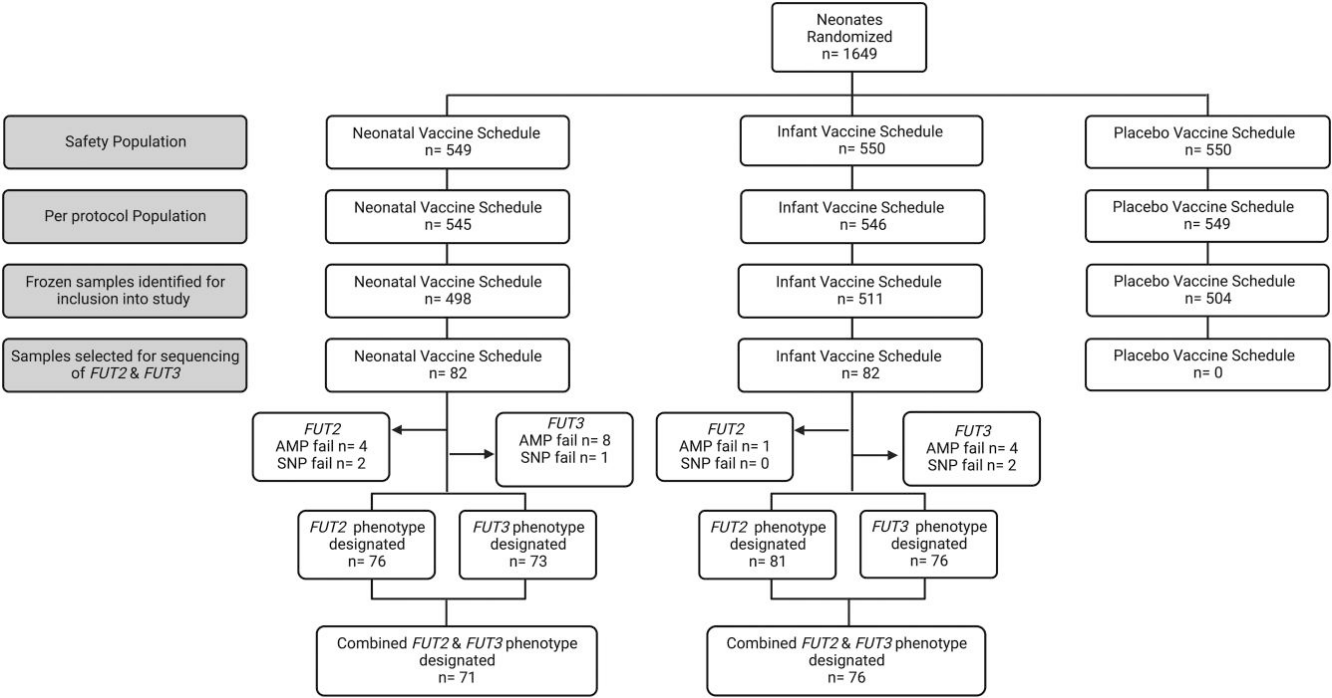
Participants through the study selection process and summary of stool specimen analysis to determine *FUT2* and *FUT3* genotype and inferred phenotype. Participant genotype was successfully determined for *FUT2* (157/164) and *FUT3* (149/164) with the combined *FUT2* and *FUT3* genotypes determined and phenotype inferred (147/164). Participants were excluded from analysis if the *FUT2* or *FUT3* amplification failed (AMP fail) or the resulting sequencing was of poor quality and not all single-nucleotide polymorphisms could be resolved (SNP fail).

The study design and recruitment for the phase IIb trial of the RV3-BB vaccine for efficacy, safety, and immunogenicity has been described in detail [33] (ACTRN12612001282875). Briefly, the study was a randomized, double-blind, placebo-controlled trial involving 1649 participants and was conducted in primary health centers and hospitals in Klaten, Central Java, and Sleman, Yogyakarta, Indonesia, from January 2013 to July 2016. Eligible infants (healthy full-term babies aged 0–5 days; birth weight, 2.5–4.0 kg) were randomized into 1 of 3 groups: neonatal vaccine, infant vaccine, or placebo. The phase IIb study was approved by the ethics committees at Universitas Gadjah Mada, Indonesia, and The Royal Children's Hospital, Melbourne, Australia. Written informed consent was provided by the parent/guardian for future evaluation studies. This study utilized samples collected from a subset (n = 164) in the per-protocol analysis population (Figure 1), with participants selected by sample availability [33].

### Stool Sample Collection and Processing

Stool samples were collected by the participant's parent/guardian, stored at 2 to 10 °C within 4 hours of collection, and transported to the Universitas Gadjah Mada microbiology laboratory within 24 hours. Upon receipt, stool samples were aliquoted and stored at −70 °C until shipped to Murdoch Children's Research Institute, Australia.

### DNA Extraction From Stool

Total DNA was extracted from 200 mg of stool with the DNeasy PowerSoil Pro Kit (QIAGEN) according to the manufacturer's instructions, with the following modifications. Samples were homogenized at 2000 rpm for 30 seconds, paused for 30 seconds, then homogenized again at 2000 rpm for 30 seconds with the Mini-Beadbeater (Biospec Products). DNA was eluted in 55 μL of solution C6. Eluted DNA was stored at −30 °C.

### Amplification of *FUT2* and *FUT3*

The enzyme-coding regions of the *FUT2* and *FUT3* genes were amplified by polymerase chain reaction (PCR) as previously described [34, 35] with the PrimeSTAR GXL DNA polymerase kit (Takara).

### *FUT2* and *FUT3* Genotyping

The *FUT2* and *FUT3* amplicons were purified with AMPure magnetic beads (Beckman Coulter) and pooled in equimolar ratios for each participant. Libraries were prepared with the Nextera XT DNA Library Preparation Kit (Illumina), pooled in equimolar ratios, and sequenced on a NovaSeq SP Lane (300 cycles, 150-bp pair-ended reads; Illumina) at the Australian Genome Research Facility.

The generated paired-end FASTQ files were screened for quality with FastQC version 0.11.9. The human reference genome assembly GRCh38 was indexed with SAMtools version 1.5, and reads were mapped against this reference genome with the Burrows-Wheeler Alignment tool version 0.7.17 [36] implemented with the *mem* algorithm. The SAM files were converted to BAM files and sorted with SAMtools version 1.9. The single-nucleotide polymorphisms (SNPs) were then called with the SAMtools *mpileup* function and the *call* function implemented in BCFtools version 1.12 [37]. SNPs were subsequently filtered and annotated with BCFtools.

### Deducing Lewis and Secretor Status

The SNPs identified in the coding regions of the *FUT2* and *FUT3* genes were investigated with the dbSNP database (https://www.ncbi.nlm.nih.gov/snp/) and the outcome predicted with the SIFT and PolyPhen tools implemented in ENSEMBL (https://asia.ensembl.org/).

Several *FUT2* SNPs were identified; participants homozygous for the c.604A>T (rs1800028) missense SNP were designated as nonsecretors. Participants homozygous for the c.418A>T (rs1047781) missense SNP were designated as weak secretors. Participants who were wild type, heterozygous, or homozygous for these SNPs were designated as secretors due to the mutation resulting in a synonymous variant: c.390C>T (rs281377), c.513C>T (rs1800027), c.525G>C (rs548254465), and c.567G>A (rs763569272). Participants who were wild type or heterozygous for these SNPs were designated as secretors: c.335C>T (rs200157007), c.556C>T (rs2032567861), c.602G>A (rs572832908), c.661C>T (rs1800029), and c.882G>A (rs1800030).

Several *FUT3* SNPs were identified; participants homozygous for c.1067T>A (rs3894326) and c.508G>A (rs3745635) missense SNPs were designated as Lewis negative, while participants who were wild type or heterozygous for these SNPs were designated as Lewis positive. Participants who were wild type or heterozygous for c.612A>G (rs28362465), c.314C>T (rs778986), c.290G>A (rs780009544), and c.202T>C (rs812936) were also designated as Lewis positive.

### Determination of RV3-BB Vaccine Response

A blood sample was collected as previously described [33]. Serum was frozen at −70 °C and shipped to the laboratory at Murdoch Children's Research Institute. Serum anti-rotavirus immunoglobulin A (IgA) antibody titers were measured with previously described methods [32, 33]. The shedding of RV3-BB vaccine–like virus in stool was detected with a rotavirus VP6–specific reverse transcription PCR assay and confirmed by sequence analysis as previously described [31, 33].

Cumulative vaccine take was defined as a serum immune response (ie, a ≥3-fold increase in titer from baseline) of anti-rotavirus IgA or serum-neutralizing antibodies (SNAs) 28 days following dose administration or detection of RV3-BB virus excretion by reverse transcription PCR analysis of stool specimens at least once during days 3 to 7 following dose administration, as previously described [33].

### Statistical Analysis

The frequencies of positive cumulative vaccine take by Lewis status, secretor status, and combined Lewis and secretor status were expressed as proportions and percentages. Analysis was performed on the infant and neonatal vaccine groups combined, where appropriate. Statistical analysis was performed with Stata (version 17; StataCorp). Study outputs of cumulative vaccine take, SNA response, serum IgA response, and RV3-BB shedding were analyzed with respect to host HBGA phenotype through relative risk (RR) analysis via the χ^2^ test. *P* values <.05 were considered significant. If <5 participants were reported in any phenotype, these data were excluded from statistical analysis.

## RESULTS

### Participant Demographics

The demographics for the participants in this study were comparable to those previously described for the per-protocol population (Supplementary Table 1) [33].

### *FUT2* and *FUT3* Genotypes and Inferred Phenotypes

For *FUT2*, all SNPs under investigation could be determined and phenotype inferred for 157 of 164 participants: gene amplification failed for 5 participants, and 2 had poor sequencing quality preventing SNP determination. SNP frequencies were determined for the 157 participants. Two participants (1.2%) were homozygous for the missense variant c.604C>T (rs1800028) and were designated as nonsecretors (Table 1). Forty participants (24.4%) were homozygous for the missense variant c.418A>T (rs1047781) and were designated as weak secretors. The remaining 115 (70.1%) were wild type or heterozygous for the SNPs identified and designated as secretors. The distribution of *FUT2* SNPs and the allele frequencies detected is shown in Supplementary Table 2.

**Table 1.** Distribution of Lewis and Secretor Phenotypes

	Vaccine Schedule, No. (%)		
	Neonatal	Infant	Combined
Secretor phenotype	76	81	157
Secretor	55	60	115 (73.2)
Nonsecretor	0	2	2 (1.3)
Weak secretor	21	19	40 (25.5)
Lewis phenotype	73	76	149
Positive	65	63	128 (85.9)
Negative	8	13	21 (14.1)
Combined phenotype	71	76	147
*Le, Se*	45	49	94 (63.9)
*Le, se*	0	1	1 (0.7)
*Le, Se^w^*	18	13	31 (21.1)
*le, Se*	7	9	16 (10.9)
*le, Se^w^*	1	4	5 (3.4)
*le, se*	0	0	0 (0)

Abbreviations: *le*, nonfunctional *FUT3* (Lewis negative); *Le*, at least 1 functional gene encoding *FUT3* (Lewis positive); *se*, nonfunctional *FUT2* (nonsecretor); *Se*, at least 1 functional gene encoding *FUT2* (secretor); *Se^w^*, weak secretor.

For *FUT3*, all SNPs under investigation could be determined and phenotype inferred for 149 of 164 participants: gene amplification failed for 12 participants, and 3 had poor sequencing quality preventing SNP determination (Figure 1). Overall, 21 (14.1%) of 149 participants were determined to be Lewis negative (Table 1). Of these, 17 were homozygous for the missense variant c.1067T>A (rs3894326), and 4 were homozygous for missense variant c.170G>S (rs3745635). The remaining 128 (85.9%) were wild type or heterozygous for these SNPs and designated as Lewis positive. The distribution of *FUT3* SNPs and the allele frequencies detected is shown in Supplementary Table 3.

Overall, the combined phenotype could be determined for 147 participants, with 94 (63.9%) designated as Lewis-positive secretors, 1 (0.7%) as a Lewis-positive nonsecretor, and 31 (21.1%) as Lewis-positive weak secretors. A further 16 (10.9%) were designated as Lewis-negative secretors and 5 (3.4%) as Lewis-negative weak secretors (Table 1).

Except for the nonsecretor phenotype, which was observed in only 2 participants in the infant schedule, the proportion of each phenotype was similar between the neonatal and infant schedules (Table 1).

### Vaccine Take Stratified by Lewis and Secretor Status

Cumulative vaccine take reported in the trial populations was high: 94% in the neonatal schedule and 99% in the infant schedule [33]. Positive vaccine take was recorded for 144 of 147 participants with the combined phenotype determined (97.9%). The participants without vaccine take were in the neonatal group, and their determined phenotypes were Lewis-positive secretor (n = 2) and Lewis-negative secretor (n = 1).

Given that the demographics and the HBGA genotypes were comparable between the participants from the neonatal and infant schedules, the data were combined for subsequent analysis. Cumulative vaccine take did not have evidence for a strong association with participant secretor status (RR, 1.00 [95% CI, .94–1.06]; *P* = .97), Lewis phenotype (RR, 1.03 [95% CI, .94–1.14]; *P* = .33), or combined secretor/Lewis phenotype: Lewis-positive weak secretor (RR, 0.98 [95% CI, .95–1.01]; *P* = .41), Lewis-negative secretor (RR, 1.04 [95% CI, .92–1.19]; *P* = .35), Lewis-negative weak secretor (RR, 0.98 [95% CI, .95–1.01]; *P* = .74) (Table 2). Nor was a difference observed when analyzed according to each component of vaccine take: SNA response, serum IgA response, combined SNA/serum IgA response, or stool excretion of vaccine-like virus.

**Table 2.** Positive Vaccine Take and Components of Vaccine Take According to Secretor and Lewis Antigen Phenotype Status

	Positive Vaccine Take			Positive for Serum IgA and/or SNA Response			Positive for Serum IgA Response			Positive for SNA Response			Stool Shedding Vaccine-like Virus		
Phenotype	No. (%)	RR (95% CI)	*P* Value	No. (%)	RR (95% CI)	*P* Value	No. (%)	RR (95% CI)	*P* Value	No. (%)	RR (95% CI)	*P* Value	No. (%)	RR (95% CI)	*P* Value
Secretor (n = 157)															
Secretor	112/115 (97.4)	1 [Ref]		96/115 (83.5)	1 [Ref]		91/115 (79.1)	1 [Ref]		37/115 (32.2)	1 [Ref]		81/115 (70.4)	1 [Ref]	
Nonsecretor	2/2 (100)	…		2/2 (100)	…		2/2 (100)	…		2/2 (100)	…		2/2 (100)	…	
Weak secretor	39/40 (97.5)	1.00 (.94–1.06)	.97	36/40 (90.0)	0.93 (.81–1.06)	.32	33/40 (82.5)	0.96 (.81–1.14)	.65	17/40 (42.5)	0.76 (.48–1.18)	.24	31/40 (77.5)	0.91 (.74–1.12)	.39
Lewis (n = 149)															
Positive	126/128 (98.4)	1 [Ref]		109/128 (85.2)	1 [Ref]		102/128 (79.7)	1 [Ref]		46/128 (35.9)	1 [Ref]		95/128 (74.2)	1 [Ref]	
Negative	20/21 (95.2)	1.03 (.94–1.14)	.33	18/21 (85.7)	0.99 (.82–1.20)	.95	17/21 (81.0)	0.98 (.79–1.23)	.89	7/21 (33.3)	1.08 (.56–2.06)	.82	14/21 (66.7)	1.11 (.81–1.53)	.47
Combined (n = 147)															
*Le, Se*	92/94 (97.9)	1 [Ref]		78/94 (83.0)	1 [Ref]		73/94 (77.7)	1 [Ref]		31/94 (33.0)	1 [Ref]		68/94 (72.3)	1 [Ref]	
*Le, Se^w^*	31/31 (100)	0.98 (.95–1.01)	.41	28/31 (90.3)	0.92 (.79–1.06)	.32	26/31 (83.9)	0.93 (.77–1.12)	.46	13/31 (41.9)	0.79 (.47–1.30)	.37	24/31 (77.4)	0.93 (.74–1.17)	.58
*Le, se*	1/1 (100)	…	.88	1/1 (100)	…		1/1 (100)	…		1/1 (100)	…		1/1 (100)	…	
*le, Se*	15/16 (93.8)	1.04 (.92–1.19)	.35	13/16 (81.3)	1.02 (.79–1.31)	.87	13/16 (81.3)	0.96 (.74–1.24)	.75	4/16 (25)	1.32 (.54–3.23)	.53	10/16 (62.5)	1.16 (.78–1.73)	.42
* le, Se^w^*	5/5 (100)	0.98 (.95–1.01)	.74	5/5 (100)	0.83 (.76–.91)	.31	4/5 (80)	0.97 (.62–1.52)	.90	3/5 (60)	0.55 (.25–1.19)	.22	4/5 (80)	0.90 (.57–1.43)	.71
*le, se*	0	…		0	…		0	…		0	…		0	…	

Phenotypes were inferred from genotype data. See Methods for descriptions of secretor and Lewis phenotypes.

Abbreviations: IgA, immunoglobulin A; *le*, nonfunctional *FUT3* (Lewis negative); *Le*, at least 1 functional gene encoding *FUT3* (Lewis positive); Ref, reference; RR, relative risk; *se*, nonfunctional *FUT2* (nonsecretor); *Se*, at least 1 functional gene encoding *FUT2* (secretor); *Se^w^*, weak secretor; SNA, serum-neutralizing antibody.

Secretors were less likely to shed RV3-BB vaccine strain in stool than to have a serum response (SNA and/or serum IgA [sIgA] response) (RR, 0.84 [95% CI, .73–.97]; *P* = .0188) (Supplementary Table 4). However, no difference was observed for weak secretors to either shed RV3-BB vaccine strain in the stool or have a serum response (SNA and/or sIgA response) (RR, 0.86 [95% CI, .71–1.05]; *P* = .13). Similarly, participants who were Lewis positive were less likely to shed RV3-BB vaccine strain in the stool than to exhibit a positive SNA/sIgA response (RR, 0.87 [95% CI, .77–.98]; *P* = .03); yet, for individuals who were Lewis negative, no difference was observed between rates of shedding and/or SNA/sIgA response (RR, 0.78 [95% CI, .55–1.10]; *P* = .15).

## DISCUSSION

This study provides further evidence that the human neonatal RV3-BB vaccine produced positive cumulative vaccine take, regardless of participant HBGA status. These findings build on the previous study from the phase IIa trial in NZ, which also found no difference in positive vaccine take by secretor status, Lewis status, or combined Lewis and secretor status [34].

Our previous report was limited due to the relatively small sample size (n = 46) in a primarily Caucasian population from NZ [34]. In both populations, the most prevalent phenotype was Lewis-positive secretor (60.9% in NZ study vs 63.9% in this study). However, these populations differed in the relative frequencies of other HBGA phenotypes. In the NZ study, 26.1% of participants were Lewis-positive nonsecretors vs a single participant in the current study (0.7%). A frequent phenotype in this study, Lewis-positive weak secretor, was not observed in the NZ cohort, which is to be expected given the rarity of this phenotype in Caucasian populations [7, 8]. Despite the differences in HBGA status in these 2 distinct populations, no impact was observed in RV3-BB vaccine response, suggesting that HBGA status is not a restriction factor for the RV3-BB vaccine strain. Neither study reported the rare Lewis-negative nonsecretor phenotype, which limited the ability to extend the conclusion to this rare phenotype.

Few studies have reported HBGA frequencies in Indonesian populations. The SNPs described in this study were consistent with prior studies and add to the description of HBGA status of the Javanese population. The pattern of *FUT2* mutations were similar to previous studies in Indonesia that identified variant c.390C>T (rs281377, secretors) at a frequency of 89.3% as compared with 80.3% in our study and c.604A>T (rs1800028, nonsecretors) at 3.3% vs 1.3% in our study [38]. The Le(a+b+) phenotype was first described in an Indonesian family [39], and the frequency of the c.418A>T variant (rs1047781, weak secretors) has been cited between 20% and 48.5% as compared with 25.4% in our study [38]. The c.1067T>A variant (rs3894326, Lewis negative) was also first described from blood donors in Jakarta, Indonesia, at a relatively high incidence (30%) [40], whereas this study revealed a lower frequency of 11.2%. A missense mutation c.59T>G previously found in the Indonesian population was not detected in our cohort [40].

In the absence of a serologic correlate for rotavirus infection, most vaccine trials have used IgA seroconversion as a surrogate marker of protection. Higher rates of Rotarix vaccine response (seropositivity) measured by serum IgA levels were noted in secretors as compared with nonsecretors in studies conducted of infants in Pakistan [24], Ghana [41], and Nicaragua [13]. In the Nicaraguan study, IgA seroconversion was not observed in Lewis-positive nonsecretors, with seroconversion observed in 26% of Lewis-positive secretors and 32% of individuals who were Lewis negative [13]. Limited studies have investigated whether HBGA status contributes to differences in vaccine strain shedding in the stool, vaccine take, or vaccine effectiveness for the P[8]-based vaccines. HBGA secretor status was associated with differences in the rate of Rotarix vaccine virus shedding in South Africa but not in Malawi. In the South African study, 77% of infants who shed vaccine virus were secretors, whereas there were no secretors in the group that did not shed [27]. These data from clinical trials contrast with data linking HBGA status with vaccine efficacy or failure in low- and middle-income countries. A decreased risk of vaccine failure with a P[8] vaccine (Rotarix) was observed in nonsecretors and Lewis-negative phenotypes if the infecting genotype was P[8] or P[4] in Kenya, Mali, and Ghana, but no association was found for P[6] infections [25]. In Malawi, decreased P[8] vaccine (Rotarix) failure was also observed in nonsecretors in association with P[8] and P[4] infections, but no difference was observed for Lewis status. In this Malawi study, participants who were Lewis negative had a higher risk of vaccine failure with P[6] infections [20]. But in Bangladesh, no difference in vaccine efficacy was observed between secretors and nonsecretors after administration of Rotarix [29]. The inconsistent outcomes from these studies may reflect differences in study methodology and vaccine constructs, regional genotypic differences in circulating rotavirus strains, and differences in characteristics of HBGA within the study population.

The P[6] genotype is less prevalent globally than P[8] and P[4] but is regionally dominant in Africa [42]. A unique feature of the P[6] genotype is the detection of naturally attenuated variants isolated from neonates [30]. The RV3-BB vaccine is derived from a naturally attenuated neonatal G3P[6] strain [30]. The nature of attenuation is not well understood, and naturally attenuated neonatal P[6] variants are genetically distinct from those associated with infections in infants and older children [43]. Several amino acid changes have been observed in neonatal variants at the basal surface of the VP8*. Furthermore, crystal structures of neonatal P[6] VP8* alone and in complex with H-type I HBGA (equivalent to antigen expressed by Lewis-negative secretors) revealed subtle structural changes in the binding sites that may restrict its ability to bind to branched glycans [44]. These studies provide a structural basis for the age-related tropism of these P[6] variants, as developmentally regulated unbranched glycans are more abundant in the neonatal gut [43, 44].

Limited studies have investigated P[6] wild type strains in relation to HBGA status. In a Korean study, 3 of 5 neonates with P[6] infection were Lewis negative and 2 of 5 were weak Lewis positive [28]. A study in Burkina Faso revealed that wild type P[6] rotaviruses mainly infect children who are Lewis negative but could infect those who are Lewis positive and negative regardless of secretor status [18]. Similarly, a study in Malawi revealed that the Lewis-negative phenotype was more common in infants with genotype P[6] strain infection vs infection with P[8] or P[4] strains [20]. Furthermore, population-level differences in the polymorphisms of *FUT2* and *FUT3* may affect the distribution of P[6] strains; the frequency of the Lewis-negative phenotype in African populations may in part contribute to the high circulation of P[6] strains observed in Africa and some South American countries [18, 42, 45]. The prior study of infants from the phase IIa trial in NZ reported that Lewis positivity was not a restriction factor for the RV3-BB vaccine, with 37 of 40 (95.2%) who were Lewis positive and 3 of 3 (100%) who were Lewis negative demonstrating positive vaccine take [34]. In this study, 126 (98.4%) of 128 participants who were Lewis positive and 20 (95.2%) of 21 who were Lewis negative demonstrated vaccine take, further supporting that Lewis positivity is not a restriction factor for the RV3-BB vaccine.

A strength of this study is that the participants' genotypes were determined by sequencing and the phenotypes subsequently inferred by detection of known SNPs. This method is preferable to the phenotype being inferred from PCR/restriction fragment length polymorphism analysis, which is limited to identifying the *FUT2* mutation G428A.

There are some limitations of this study. There was still a small number of participants who were Lewis-positive nonsecretors and Lewis-negative weak secretors, with no Lewis-negative nonsecretors. Additionally, due to the high rate of vaccine take (94% in the neonatal schedule and 99% in the infant schedule), there was a small number of participants without vaccine take, limiting our ability to examine HBGA status associated with the absence of vaccine take [33].

In conclusion, this study demonstrated that administration of the RV3-BB human neonatal vaccine produced a positive cumulative vaccine take regardless of HBGA status. No significant difference in positive vaccine take was observed by secretor status, Lewis status, or Lewis and secretor status combined. This study provides further evidence that vaccine take of the RV3-BB vaccine is not affected by population-level differences in HBGA status and thus may be a useful asset in combatting location-dependent challenges to effective rotavirus vaccine coverage.

## Supplementary Data

Supplementary materials are available at *The Journal of Infectious Diseases* online. Consisting of data provided by the authors to benefit the reader, the posted materials are not copyedited and are the sole responsibility of the authors, so questions or comments should be addressed to the corresponding author.

## Supplementary Material

jiad351_Supplementary_Data

## Notes

***Acknowledgments.*** We sincerely thank the infants and their families for participating in the RV3 study. We also thank the members of the Universitas Gadjah Mada Paediatric Research Office and Microbiology Laboratory, as well as the site coordinators, midwives, and hospital staff, who assisted in the RV3 trial. We thank Karen Boniface, who assisted in data interpretation.

***Author contributions.*** Study conception and design: J. E. B., A. H., Y. S. Data acquisition and analysis: E. W., D. P., D. S. O., N. B.-S., E. A. L., S. G. B., A. H., C. M. D., J. A. T., C. D. S., H. N. C. M. D. wrote the manuscript. All authors approved the final version of the manuscript.

***Data availability.*** Deidentified group data may be available for sharing on application via the corresponding author. This application must include the relevant proposal detailing the intended use of the data, the ethics approval for this proposal, and a signed data-sharing agreement. Additional study documents, including the study protocol, statistical analysis plan, and informed consent, are available on application via the corresponding author on publication of this report.

***Financial support*.** This work was supported by the Bill & Melinda Gates Foundation (OPP1127967 and OPP1111055), the National Health and Medical Research Council (491239), and PT Bio Farma. This research at Murdoch Children's Research Institute was supported by the Victorian Government’s Operational Infrastructure Support Program. C. M. D. was supported through the Australian National Health and Medical Research Council with an Early Career Fellowship (1113269). Funding to pay the Open Access publication charges for this article was provided by the Bill & Melinda Gates Foundation.
