# Supplementary material for: Vaccine Take of RV3-BB Rotavirus Vaccine Observed in Indonesian Infants Regardless of HBGA Status
Source: J Infect Dis. 2023 Aug 18;229(4):1010–8. doi: 10.1093/infdis/jiad351 (PMC11011179; doi:10.1093/infdis/jiad351)
Supplement: jiad351_Supplementary_Data [file jiad351_supplementary_data.zip › JID-77524_DONATO_Supplementary Table S2.docx]

**Supplementary Table S2. *FUT2* Distribution of SNPs and the allele frequencies**

|  |  |  | **SNP** | | |
| --- | --- | --- | --- | --- | --- |
|  |  |  | **Wildtype** | **Heterozygous** | **Homozygous** |
| **Nucleotide**  **position^** | **SNP**  **reference** | **Variant**  **type** | **n (%)** | **n (%)** | **n (%)** |
| 335 (302) | rs200157007 | Missense | CC | CT | TT |
|  |  |  | 156 (99.4) | 1 (0.6) | 0 (-) |
| 390 (357) | rs281377 | Synonymous | CC | CT | TT |
|  |  |  | 3 (1.9) | 28 (17.8) | 126 (80.3) |
| 418 (385) | rs1047781 | Missense | AA | AT | TT |
|  |  |  | 50 (31.8) | 67 (42.6) | 40 (25.4) |
| 513 (480) | rs1800027 | Synonymous | CC | CT | TT |
|  |  |  | 153 (97.5) | 4 (2.5) | 0 (-) |
| 525 (492) | rs548254465 | Synonymous | GG | GC | CC |
|  |  |  | 156 (99.4) | 1 (0.6) | 0 (-) |
| 556 (523) | rs2032567861 | Stop Gained | CC | CT | TT |
|  |  |  | 155 (98.7) | 2 (1.3) | 0 (-) |
| 567 (534) | rs763569272 | Synonymous | GG | GA | AA |
|  |  |  | 155 (98.7) | 2 (1.3) | 0 (-) |
| 602 (569) | rs572832908 | Missense | GG | GA | AA |
|  |  |  | 156 (99.4) | 1 (0.6) | 0 (-) |
| 604 (571) | rs1800028 | Stop Gained | CC | CT | TT* |
|  |  |  | 148 (94.3) | 7 (4.5) | 2 (1.3) |
| 661 (628) | rs1800029 | Stop Gained | CC | CT | TT |
|  |  |  | 156 (99.4) | 1 (0.6) | 0 (-) |
| 882 (849) | rs1800030 | Stop Gained | GG | GA | AA |
|  |  |  | 155 (97.5) | 4 (2.5) | 0 (-) |

The *FUT2* gene was successfully amplified and sequenced for 157/164 participants with the frequencies of SNPs outlined. Homozygous variants are shaded in grey.

* Homozygous likely leading to null phenotype

^ A discrepancy of 33 base pairs was consistently observed between the nucleotide position of each *FUT2* SNP obtained in this study using human reference genome assembly GRCh38.p13 (NC_000019.10) and the SNP positions published in prior studies due to the use of a different reference genome. The SNP position published in some prior literature is provided in brackets.

Abbreviation: SNP, Single nucleotide polymorphism
