# Supplementary material for: Vaccine Take of RV3-BB Rotavirus Vaccine Observed in Indonesian Infants Regardless of HBGA Status
Source: J Infect Dis. 2023 Aug 18;229(4):1010–8. doi: 10.1093/infdis/jiad351 (PMC11011179; doi:10.1093/infdis/jiad351)
Supplement: jiad351_Supplementary_Data [file jiad351_supplementary_data.zip › JID-77524_DONATO_Supplementary Table S3.docx]

**Supplementary Table S3. *FUT3* Distribution of SNPs and the allele frequencies**

|  |  |  | **SNP (reverse strand)** | | |
| --- | --- | --- | --- | --- | --- |
|  |  |  | **Wildtype** | **Heterozygous** | **Homozygous** |
| **Nucleotide**  **position** | **SNP reference** | **Variant type** | **n (%)** | **n (%)** | **n (%)** |
| 202 | rs812936 | Missense | TT | TC | CC |
|  |  |  | 147 (98.7) | 2 (1.3) | 0 (-) |
| 290 | rs780009544 | Missense | GG | GA | AA |
|  |  |  | 148 (99.3) | 1 (0.7) | 0 (-) |
| 314 | rs778986 | Missense | CC | CT | TT |
|  |  |  | 147 (98.7) | 2 (1.3) | 0 (-) |
| 508 | rs3745635 | Missense | GG | AG | AA* |
|  |  |  | 111 (74.5) | 34 (22.8) | 4 (2.7) |
| 612 | rs28362465 | Synonymous | AA | AG | GG |
|  |  |  | 147 (98.7) | 2 (1.3) | 0 (-) |
| 1067 | rs3894326 | Missense | TT | TA | AA* |
|  |  |  | 72 (48.3) | 60 (40.3) | 17 (11.2) |

The *FUT3* gene was successfully amplified and sequenced for 149/164 participants with the frequencies of SNPs outlined. Homozygous variants are shaded in grey.

* Homozygous likely leading to null phenotype

Abbreviation: SNP, Single nucleotide polymorphism
