# Supplementary material for: Vaccine Take of RV3-BB Rotavirus Vaccine Observed in Indonesian Infants Regardless of HBGA Status
Source: J Infect Dis. 2023 Aug 18;229(4):1010–8. doi: 10.1093/infdis/jiad351 (PMC11011179; doi:10.1093/infdis/jiad351)
Supplement: jiad351_Supplementary_Data [file jiad351_supplementary_data.zip › JID-77524_DONATO_Supplementary Table S4.docx]

|  | **Secretor** | | | | |  | **Weak secretor** | | | | |  | | **Lewis positive** | | | | |  | **Lewis negative** | | | | |
| --- | --- | --- | --- | --- | --- | --- | --- | --- | --- | --- | --- | --- | --- | --- | --- | --- | --- | --- | --- | --- | --- | --- | --- | --- |
|  | **Participants** | **yes** | **no** | **RR**  **(95% CI)** | **P** |  | **Participants** | **yes** | **no** | **RR**  **(95% CI)** | **P** |  | **Participants** | | **yes** | **no** | **RR**  **(95% CI)** | **P** |  | **Participants** | **yes** | **no** | **RR**  **(95% CI)** | **P** |
| **Shedding**  **VLV** | 81/115 | 81 | 34 | ref |  |  | 31/40 | 31 | 9 | ref |  |  | 95/128 | | 95 | 33 | ref |  |  | 14/21 | 14 | 7 | ref |  |
| **SNA** | 37/115 | 37 | 78 | 2.19  (1.64 - 2.93) | **<0.001** |  | 17/40 | 17 | 23 | 1.82  (1.23 - 2.71) | **0.0014** |  | 46/128 | | 46 | 82 | 2.07  (1.60 - 2.66) | **<0.001** |  | 7/21 | 7 | 14 | 2.0  (1.02 - 3.93) | **0.0308** |
| **sIgA** | 91/115 | 91 | 24 | 0.89  (0.75 - 1.04) | 0.13 |  | 33/40 | 33 | 7 | 0.94  (0.75 - 1.17) | 0.58 |  | 102/128 | | 102 | 26 | 0.93  (0.814 - 1.07) | 0.299 |  | 17/21 | 17 | 4 | 0.82  (0.57 - 1.19) | 0.292 |
| **SNA/sIgA** | 96/115 | 96 | 19 | 0.84  (0.73 - 0.97) | **0.0188** |  | 36/40 | 36 | 4 | 0.86  (0.71 - 1.05) | 0.1297 |  | 109/128 | | 109 | 19 | 0.87  (0.77 - 0.98) | **0.0296** |  | 18/21 | 18 | 3 | 0.78  (0.55 - 1.10) | 0.1473 |
|  |  |  |  |  |  |  |  |  |  |  |  |  |  | |  |  |  |  |  |  |  |  |  |  |
| **SNA** | 37/115 | 37 | 78 | ref |  |  | 17/40 | 17 | 26 | ref |  |  | 46/128 | | 46 | 82 | ref |  |  | 7/21 | 7 | 14 | ref |  |
| **sIgA** | 91/115 | 91 | 24 | 0.41  (0.31 - 0.54) | **<0.001** |  | 33/40 | 33 | 7 | 0.48  (0.32 - 0.71) | **0.0001** |  | 102/128 | | 102 | 26 | 0.45  (0.35 - 0.58) | **<0.001** |  | 17/21 | 17 | 4 | 0.41  (0.22 - 0.78) | **0.0018** |
|  |  |  |  |  |  |  |  |  |  |  |  |  |  | |  |  |  |  |  |  |  |  |  |  |
| **Vaccine-take** | 112/115 | 112 | 3 | ref |  |  | 39/40 | 39 | 1 | ref |  |  | 126/128 | | 126 | 2 | ref |  |  | 20/21 | 20 | 1 | ref |  |
| **Shedding**  **VLV** | 81/115 | 81 | 34 | 1.38  (1.22 - 1.56) | **<0.001** |  | 31/40 | 31 | 9 | 1.26  (1.06 - 1.50) | **0.0068** |  | 95/128 | | 95 | 33 | 1.33  (1.19 - 1.48) | **<0.001** |  | 14/21 | 14 | 7 | 1.43  (1.04 - 1.96) | **0.0184** |
| **SNA/sIgA** | 96/115 | 96 | 19 | 1.17  (1.07 - 1.27) | **0.0003** |  | 36/40 | 36 | 4 | 1.08  (0.97 - 1.21) | 0.1659 |  | 109/128 | | 109 | 19 | 1.16  (1.07 - 1.25) | **0.0001** |  | 18/21 | 18 | 3 | 1.11  (0.91 - 1.36) | 0.2931 |

**Supplementary Table 4. Comparison of the components of vaccine take according to secretor and Lewis antigen phenotype status**

Abbreviations: RR – relative risk, VLV – vaccine-like virus, SNA – serum neutralisation assay, sIgA – serum immunoglobulin A

Given less than five non-sectors were observed, this phenotype was excluded from statistical analysis.
