# Supplementary material for: Vaccine Take of RV3-BB Rotavirus Vaccine Observed in Indonesian Infants Regardless of HBGA Status
Source: J Infect Dis. 2023 Aug 18;229(4):1010–8. doi: 10.1093/infdis/jiad351 (PMC11011179; doi:10.1093/infdis/jiad351)
Supplement: jiad351_Supplementary_Data [file jiad351_supplementary_data.zip › JID-77524_DONATO_Supplementary Table S1.docx]

**Supplementary Table S1. Demographic characteristics**

|  | **Per protocol population** | | | **HBGA sub study** | |
| --- | --- | --- | --- | --- | --- |
|  | **Neonatal Vaccine Schedule (N=498)** | **Infant Vaccine Schedule (N=511)** | **Placebo**  **(N=504)** | **Neonatal Vaccine Schedule**  **(N=82)** | **Infant Vaccine Schedule (N=82)** |
| Age at randomization (days)* |  |  |  |  |  |
| Mean (SD) | 3.4 (1.3) | 3.5 (1.3) | 3.5 (1.3) | 3.1 (1.2) | 3.1 (1.2) |
|  |  |  |  |  |  |
| Sex |  |  |  |  |  |
| Male | 270 (54.2%) | 265 (51.9%) | 256 (50.8%) | 46 (56.1) | 45 (53.6) |
|  |  |  |  |  |  |
| Ethnicity |  |  |  |  |  |
| Javanese | 497 (99.8%) | 509 (99.6%) | 502 (99.6%) | 82 (100%) | 82 (100%) |
| Sundanese | 0 (0.0%) | 0 (0.0%) | 1 (0.2%) | 0 (0.0%) | 0 (0.0%) |
| Chinese | 0 (0.0%) | 1 (0.2%) | 0 (0.0%) | 0 (0.0%) | 0 (0.0%) |
| Malaya | 0 (0.0%) | 0 (0.0%) | 0 (0.0%) | 0 (0.0%) | 0 (0.0%) |
| Other | 1 (0.2%) | 1 (0.2%) | 1 (0.2%) | 0 (0.0%) | 0 (0.0%) |
|  |  |  |  |  |  |
| Gestational Age (weeks) |  |  |  |  |  |
| Mean (SD) | 39.55 (1.10) | 39.53 (1.08) | 39.54 (1.12) | 39.64 (1.14) | 39.46 (1.03) |
|  |  |  |  |  |  |
| Birth Weight (g) |  |  |  |  |  |
| Mean (SD) | 3117.57 (347.65) | 3136.01 (333.55) | 3106.25 (338.35) | 3083.92  (330.28) | 3135.73  (309.38) |
|  |  |  |  |  |  |
| Height/Length (cm) |  |  |  |  |  |
| Mean (SD) | 48.70 (1.65) | 48.65 (1.67) | 48.62 (1.68) | 48.68 (1.74) | 48.26 (1.99) |

***** Age at first dose of investigational product.

Calculated as (Date of Randomisation - Date of Birth)
